# Markers of enterocyte damage in celiac disease in children: is there an association with the clinical manifestations of the disease?

**DOI:** 10.3389/fped.2025.1566149

**Published:** 2025-06-12

**Authors:** Svetlana Geller, Zulhumor Umarnazarova, Noiba Azimova, Kamola Usmonova, Altinoy Kamilova

**Affiliations:** Gastroenterology Department of Republican Specialized Scientific-Practical Medical Center of Pediatrics, Ministry of Health of Republic of Uzbekistan, Tashkent, Uzbekistan

**Keywords:** celiac disease, children, fecal zonulin, intestinal fatty-acid-binding protein, enterocyte damage

## Abstract

**Actuality:**

The state of the intestinal barrier has crucial importance in the pathogenesis of celiac disease (CD). Fecal zonulin (FZ) and intestinal fatty acid binding protein (i-FABP) are important components in maintaining physiological processes in the intestine and potential biomarkers of enterocyte damage.

**Aim of study:**

To evaluate FZ and i-FABP levels as markers of small intestine injury in children with CD, depending on the clinical forms and histomorphological changes in the small intestinal mucosa.

**Materials and methods:**

In 2021–2023 yy, a single-center observational study was conducted among children with newly diagnosed CD.The level of FZ in stool and I-FABP in serum were determined using the Immundiagnostik ELISA kits (Germany).

**Results:**

Study included 75 patients,control group was 37 healthy children. The intestinal form of the CD was established in 51 (68.0%) patients,the remaining 24 (32.0%) children have CD with extraintestinal manifestations. Among children with classical CD, the mean values of FZ were 157.9 ± 29.8 ng/ml (*p* < 0.02 with control), in second group the mean values of FZ were 136.7 ± 17.0 ng/ml, (*p* < 0.05 with the control), and a statistically significant difference between the groups was *p* < 0.02. The i-FABP values in the first group were 2476.9 ± 297.4 pg/ml (*p* < 0.05 with control),and in the second −2061.47 ± 291.5 pg/ml. In the group of children with intestinal manifestations of CD, a weak positive correlation relationship was found between FZ and stool frequency (*r* = 0.35). In the second group: weak inverse correlations were between FZ and weight, and height (*r* = −0.37 and *r* = −0.36 respectively). I-FABP values in the first group moderately correlated with stool frequency (*r* = 0.53). In the group with extraintestinal manifestations, a moderate negative relationship was found between the i-FABP2 level and BMI (*r* = −0.53) and a moderate positive relationship between the i-FABP level and antibodies to tissue transglutaminase IgA (*r* = 0.58) and a weak positive correlation with histological assessment according to Marsh criteria (*r* = 0.34).

**Conclusions:**

Our study demonstrated a relationship between the clinical manifestations of CD and the levels of FZ and i-FABP. The increase in the values also can serve as marker of increased permeability and damage of the intestinal barrier, which will open up new possibilities for understanding the processes of restoration of the small intestinal mucosa.

## 1 Introduction

Celiac disease (CD) is a chronic enteropathy caused by an immune-mediated reaction to gluten that occurs in genetically predisposed people and can manifest itself at any stage of life, from early childhood to old age (1). The prevalence of CD in the world is estimated at 1.4% according to serology and 0.7% according to biopsy data, mainly among the Caucasian population (2, 3).

Intestinal villous atrophy in CD leads to malabsorption and improper digestion of nutrients, resulting in symptoms such as diarrhea, abdominal pain, weight loss, and steatorrhea. The intestinal form of CD is most common in the pediatric population and in children under 3 years of age when gluten-containing foods are introduced into the diet (4), while older children and adults are more likely to have extraintestinal signs (4, 5).

Intestinal permeability (IP) plays a decisive role in the pathogenesis of CD. The mucous membrane of the gastrointestinal tract acts as a highly specialized barrier separating the internal environment from the external, which is necessary to maintain the homeostasis of the body and prevent the entry of harmful substances and microorganisms into the circulatory system (6). Zonulin, a human protein weighing 47 kDa, plays an important role in modulating the permeability of tight junctions of the small intestine, which is fundamental for maintaining physiological processes in the intestine (7) and has become a potential non-invasive biomarker for the study of intestinal permeability. Tight junctions in CD are disrupted, allowing undigested gluten peptides to pass through the epithelial barrier, triggering an immune response involving both the adaptive and innate immune systems (8). Study by Fasano et al. (9) showed that zonulin expression in intestinal tissues increased during the acute phase of CD, a clinical condition, in which tight junctions opened and permeability increased. In most studies, zonulin is determined in two biological substances (blood and feces) (10–13). It is assumed that determination of zonulin levels in feces may be a more sensitive and specific method for assessing intestinal permeability, since it reflects protein secretion directly from the site of epithelial damage (14, 15). There are only a few studies in the literature on the determination of fecal zonulin in children with CD; we found only one study by Gallego et al, who found that the concentration of zonulin in feces was higher in children with active CD compared to healthy people and those who followed a gluten-free diet (GFD) (16).

Serum cytosolic intestinal Fatty-Acid-Binding Protein (i-FABP), which is a small 5 kDa protein that accounts for 1%–2% of the total cytosolic protein in enterocytes, is also a non-invasive marker of enterocyte injury (17). The tissue specificity of i-FABP, as well as its ability to be mea-sured in readily available noninvasive samples (e.g., urine), make it an attractive biomarker of upper GI tissue injury/damage. Previous studies have shown that patients with IBD and CD have significantly higher circulating serum i-FABP concentrations compared to healthy individuals, but the evidence has not always been specifically (18–28).

In several studies, it has been noted that the level of i-FABP in blood plasma in patients with CD is higher than in healthy people at diagnosis compared to healthy people, which indicates damage to the small intestinal mucosa (20, 21, 24, 26, 29–32). In CD in adults, it has been found that i-FABP are involved as mediators of inflammatory processes in the tissues where they are represented, and changes in the enterocytes of the epithelium disrupt the absorption of nutrients, which can lead to changes in intracellular lipid transport, protein expression (33).

Thus, a review of the literature on the concentration of zonulin and i-FABP demonstrated the limitations of studies on these indicators in children with CD depending on the clinical forms and histomorphological changes in the small intestinal mucosa.

The aim of our study was to evaluate fecal zonulin and i-FABP indicators as markers of small intestine injury in children with CD, depending on the clinical forms and histomorphological changes in the small intestinal mucosa for future assessment of their potential diagnostic and prognostic value.

## 2 Methods

### 2.1 Study subjects and grouping

In 2021–2023 yy., at the Gastroenterology Department of Republican Specialized Scientific-Practical Medical Center of Pediatrics (RSSPMCP), Tashkent, Uzbekistan a single-center prospective study was conducted.

**Inclusion Criteria**: children newly diagnosed CD aged 1 to 16 years and healthy children of the same age.

**Exclusion Criteria**: previously diagnosed with CD; exclusion of gluten from the diet at the time of selection for the study; refusal to sign informed consent to participate in the study.

The study did not include patients with comorbid conditions such as Down syndrome, Hoshimoto's thyroiditis.

Controls were recruited from the community and included healthy volunteers with no known history of gastrointestinal diseases or symptoms per Rome IV and eating gluten without restriction.

**Limitations of the study**: a small group of patients and a lack of studies in dynamics on the background of a gluten-free diet.

### 2.2 General clinical data collection

General clinical examination of patients included anamnesis, objective examination, instrumental and laboratory research methods. Objective examination was conducted according to the standard scheme. At the same time, attention was paid to the general condition, the presence of specific complaints, the time of onset of symptoms of the disease, the state of internal organs and systems, changes in the nature of the stool were taken into account. The assessment of the physical development of children was carried out according to reference tables of anthropometric indicators proposed by experts of the World Health Organization, using the WHO Anthro, WHO AnthroPlus programs (34).

### 2.3 Laboratory analysis

#### 2.3.1 Confirmation of diagnosis

To confirm the diagnosis of CD, 2020 ESPGHAN criteria were used: the first stage was the determination of antibodies to tissue transglutaminase IgA and total IgA, IgG, IgM [Orgentec Diagnostika GmbH Enzyme-Linked Immunosorbent Assay (ELISA) kit for quantitative determination in human serum, Cat. No. 416-5400A]. If the values of antibodies to tissue transglutaminase IgA increased above 100 U/ml, the patient moved on to the next stage, at which, due to the absence of endomysial antibodies in the Republic of Uzbekistan, all serologically positive patients underwent to upper gastrointestinal endoscopy and duodenal biopsy (by Pentax EG2930 K endoscope after overnight fasting). With parental consent, 51 patients underwent biopsy of the post-bulbar portion of the duodenum (4 biopsies). The biopsy samples were included in neutral buffered formalin and processed according to standard procedures, in order to be evaluated by two experienced pathologists who graded the histologic findings according to the modified Marsh criteria (35).

According to indications, examination was performed for the presence of specific heterodimers DQ2 and DQ8 (*n* = 24).

#### 2.3.2 Fecal zonulin detection methods

To assess the function of tight junctions of the small intestine, the level of fecal zonulin was determined using the IMMUNDIAGNOSTIK enzyme-linked immunosorbent assay kit (Germany, Cat. No. K 5600). The fecal extract in frozen form was stored at −20°C. The analysis is based on the competitive ELISA method, the study was carried out in duplicates with the construction of an analytical curve.

#### 2.3.3 Intestinal fatty acid-binding protein detection methods

To assess the presence of enterocyte damage, the level of intestinal fatty acid-binding protein in blood serum or plasma was determined using the IMMUNDIAGNOSTIK ELISA kit (Germany, Cat. No. K 6809).

### 2.4 Ethical statement

Approved by the Ethics Council of the Republican Specialized Scientific and Practical Medical Center of Pediatrics of the Ministry of Health of the Republic of Uzbekistan IP-2021-1223. Informed written consent was acquired from their parents or guardians and the research was conducted in compliance with the World Medical Association Declaration of Helsinki.

### 2.5 Statistical analysis

The sample size was not calculated preliminarily, a continuous study of children who came to our center with the first established CD was carried out. Studies were carried out with the written consent of their parents.

Missing data were handled using the Complete Case Analysis method, in which rows/columns containing gaps were excluded from the data set. Statistical analysis was performed using GraphPad Prism (version 9.3.1, 2021). Using statistical functions with calculation for quantitative values of the arithmetic mean (M), standard deviation (SD), median (Me), quartiles (Q1; Q3), Student's criterion (t), Mann–Whitney criterion. The normality of the distribution of quantitative variables was checked using the Kolmogorov–Smirnov method. The following indicators had a normal distribution in a group 1: weight, hemoglobin, total protein, i-FABP; in group 2: age, weight, height, age of introduction of gluten-containing products, stool frequency, age of first symptoms, tTG-IgA, hemoglobin, total protein, i-FABP.

For measuring the strength and direction of the relationship between two variables Pearson's correlation coefficient was used.

Categorical variables were expressed as absolute and relative values. 95% CI for the proportion was calculated using the Wald normal approximation method.

The differences were considered statistically significant at *p* < 0.05, the calculation was made by the two-sided *p*-value.

## 3 Results

### 3.1 Demographic and clinical characteristics

Study was conducted in 75 children. Girls were more prevalent than boys, when distributing by gender—58.6% (44). The median age was 3 years 3 months [1; 15.5 years]. Controls were 37 children from 1 to 16 years old, whose average age was 4.5 ± 1.8 years.

Intestinal or classical form of the disease was in 51 (68.0%) patients (group 1), the remaining 24 (32.0%) children were diagnosed with CD with extraintestinal manifestations (group 2). Girls prevailed in both groups. The average age between the groups did not have a statistically significant difference, however, in the group of patients with extraintestinal manifestations, there was a later introduction of gluten-containing products into the diet 12.7 ± 3.7 and 7.5 ± 5.1 months, respectively (*p* < 0.001). The time of appearance of the first complaints in both groups coincided, on average, 2.5 years, however, due to the non-specificity of symptoms and the complexity of diagnosis, the age of diagnosis of CD with extraintestinal symptoms was several times higher 65.6 ± 48.3 and 38.2 ± 28.6 months, respectively (*p* < 0.005).

The leading clinical symptoms in the group of patients with typical manifestations were abdominal distention (48–94.1%), abdominal pain (24–47.0%), vomiting (25–49.0%), diarrhea (51–100.0%) with a stool frequency of 4.5 ± 1.8 times, lag in weight (30–58.8%) and height (38–74.5%) of moderate and severe degree. Of the extraintestinal symptoms in this group, a quarter had pain in the bones and joints, caries, and every fifth person had headaches (Table 1).

**Table 1:** Comparative clinical and laboratory characteristics of patients with CD.

Characteristic	CD with intestinal manifestations (*n* = 51)	CD with extraintestinal manifestations (*n* = 24)	Control group (*n* = 37)	*p*-value
Age, Me (Q1–Q3), months	40 (28;79)	50 (32;100)	72 (42;102)	*p > 0.05 **p < 0.001 ***p > 0.05
Number of boys/girls abs. (%)	20/31 (39,2/60,8)	11/13 (45,8/54,2)	30/7 (81,1/18,9)	*p > 0.05 **p < 0.01 ***p < 0.001
Age of introduction of gluten-containing products, Me (Q1–Q3), months	6,5 (6;10)	10 (8:14)	8 (7;10)	*p < 0.001 **p > 0.05 ***p > 0.05
Age of appearance of the first signs, Me (Q1–Q3), months	24 (14;36)	24 (14,5;42)	–	*p > 0.05
Age of diagnosis, Me (Q1–Q3), months	36 (26;77)	45 (41,5;94,5)	–	*p < 0.005
Gastrointestinal symptoms				
Abdominal distention	48 (94,1%)	10 (41,6%)	–	*p < 0.001
Abdominal pain	24 (47,0%)	8 (33,3%)	–	*p > 0.05
Stool frequency, times	4 (3;5)	1 (1;2)	1 (1;2)	*p < 0.001 **p < 0.001 ***p > 0.05
Constipation	–	6 (25,0%)	–	–
Vomiting	25 (49,0%)	–	–	–
Extraintestinal symptoms				
Recurrent stomatitis	8 (15,6%)	10 (41,6%)	–	*p < 0.02
Bone pain	13 (25,5%)	11 (45,8%)	–	*p < 0.02
Caries	13 (25,5%)	10 (41,6%)	–	*p < 0.02
Headaches	10 (19,6%)	9 (37,5%)	–	*p > 0.05
Anemia	9/17,6%	12/50,0%	–	*p < 0.01
Hair loss	–	6/25,0%	–	–
Anthropometric indicators				
Height (z-score)	−2.57 (−3.28; −1.82)	−1.605 (−2.73; −1.15)	0,31 (0,11;0,52)	*p < 0.02 **p < 0.001 ***p < 0.001
Weight (z-score)	−2.125 (−3.05; −1.58)	−1.85 (−3.13; −0.645)	0,54 (0,24;0,83)	*p < 0.02 **p < 0.001 ***p < 0.001
BMI (z-score)	−0.92 (−2.02; 0.19)	−0.985 (−1.94; 0.27)	0,49 (0,3;1,0)	*p > 0.05 **p < 0.001 ***p < 0.001
Laboratory indicators				
Hemoglobin, g/L	105 (87;115)	112.5 (97;122)	118.6 (115;125)	*p > 0.05 **p < 0.001 ***p > 0.05
Total protein, g/L	56 (49;63)	62 (57;65)	72 (68; 76)	*p > 0.05 **p < 0.001 ***p < 0.001
Leukocytes 10^9^	8.05 (6.37;11.22)	8.075 (6.525; 11.215)	7.0 (4.35;10.87)	*p > 0.05 **p > 0.05 ***p > 0.05
tTG-IgA, Me (Q1–Q3), (U/ml)	174 (118;400)	166.9 (110;370,9)	5.2 (4.3;8.7)	*p > 0.05 **p < 0.001 ***p < 0.001
tTG-IgG, Me (Q1–Q3), (U/ml)	53 (11; 126.2)	23.3 (5.57; 93.8)	3.8 (2.6;4.8)	*p < 0.02 **p < 0.001 ***p < 0.001
Histological picture according to Marsh	*n* = 40	*n* = 11		
Marsh I infiltrative	0	0	–	*p > 0.05
Marsh II Hyperplastic	2/5,0%	7/63,6%	–	*p < 0.001
Marsh III Destructive:	38/95,0%	4/36,4%	–	*p < 0.002
Marsh IIIa	12/31,5%	3/75%	–	*p < 0.02
Marsh IIIb	20/52.6%	1/25%	–	*p > 0.05
Marsh IIIc	6/15,9%	–	–	–
HLA typing	*n* = 11	*n* = 13		
HLA-DQ2.5	7/63,6%	5/38,4%	–	*p > 0.05
HLA-DQ2.2	2/18,2%	5/38,4%	–	*p > 0.05
HLA-DQ7.5	2/18,2%	3/23,2%	–	*p > 0.05

* *p*-validity between groups of patients with CD with intestinal manifestations and CD with extraintestinal manifestations.

** *p*-validity between groups of patients with CD with intestinal manifestations and control group.

*** *p*-validity between groups of patients with CD with extraintestinal manifestations and control group. Counts (%), medians (Me, interquartile range) are presented appropriate.

CD, celiac disease; Me, median; tTG-IgA, tissue transglutaminase IgA; tTG-IgG, tissue transglutaminase IgG; BMI, body mass index; HLA, human leukocyte antigen system.

Whereas children from the group 2 did not have any significant complaints from the gastrointestinal tract. These patients were referred to a gastroenterologist mainly to assess the condition associated with low height (12–50.0%) and/or weight delay (13–54.2%), as well as frequent stomatitis (10–41.6%), caries (10–41.6%), hair loss (6–25.0%), refractory anemia (12–50,0%).

Analysis of anthropometric data by (z-score) showed that, on average, growth deficiency in the group 1 was −2.57 (−3.28; −1.82) and −1.605 (−2.73; −1.15), respectively, which was significantly important compared to patients with extraintestinal manifestations of CD (*p* < 0.02). There was also a significant difference between the groups in terms of weight deficit, which was −2.125 (−3.05; −1.58) and −1.85 (−3.13; −0.645), respectively (*p* < 0.02). There was no statistically significant difference in BMI indicators (Table 1).

An increased level of tTG-IgA (>10 U/ml) was found in 48 (94.1%) children with classical CD, the mean value was 230 ± 147.05 U/L. In 3 children, the level of IgA was below the age-related reference range. In the group 2, the mean value of tTG-IgA was 209.1 ± 131.5 U/ml, selective IgA deficiency was also present in 3 patient.

A statistically significant difference was observed between the groups in terms of the level of tTG-IgG, the mean value in the group 1 was 96.2 ± 20.2 U/L, in the group 2 it was 1.8 times less—53.7 ± 19.8 U/ml, *p* < 0.02 (Table 1).

With parental consent, 51 patients underwent biopsy of the post-bulbar portion of the duodenum (4 biopsies). In the assessment of morphological changes, stage I, infiltrative, was absent, stage II (hyperplastic) was observed in 9 (17.6%) children. In 42 (82.3%) children, stage III (destructive) changes in the mucous membrane of the small intestine were noted, which corresponded to the clinical picture of the disease.

For patients with CD with extraintestinal manifestations, hyperplastic changes in the small intestinal mucosa Marsh II were more characteristic compared to the group 1 (63.6% and 5.0%, respectively, *p* < 0.001). Among children with classical CD, the destructive picture according to Marsh IIIb prevailed (20–52.6%), in the group 2—Marsh IIIa in 75%, *p* < 0.02 (Table 1).

12 patients (50.0%) were carriers of HLA-DQ2.5, encoded by the genes DQA1_05 (alpha- chain) and DQB1_02 (beta- chain), 7 (29.1%) were carriers of HLA-DQ2.2, encoded by the genes HLA-DQ2.2 (DQA1 * 02/DQB1 * 02) and 5 (20.8%)—DQ7.5, encoded by the genes (DQA1 * 05, DQB1 * 03: 01). DQ 8 was not detected in any case.

### 3.2 Fecal zonulin levels depending on the form of celiac disease

**Figure 1:**
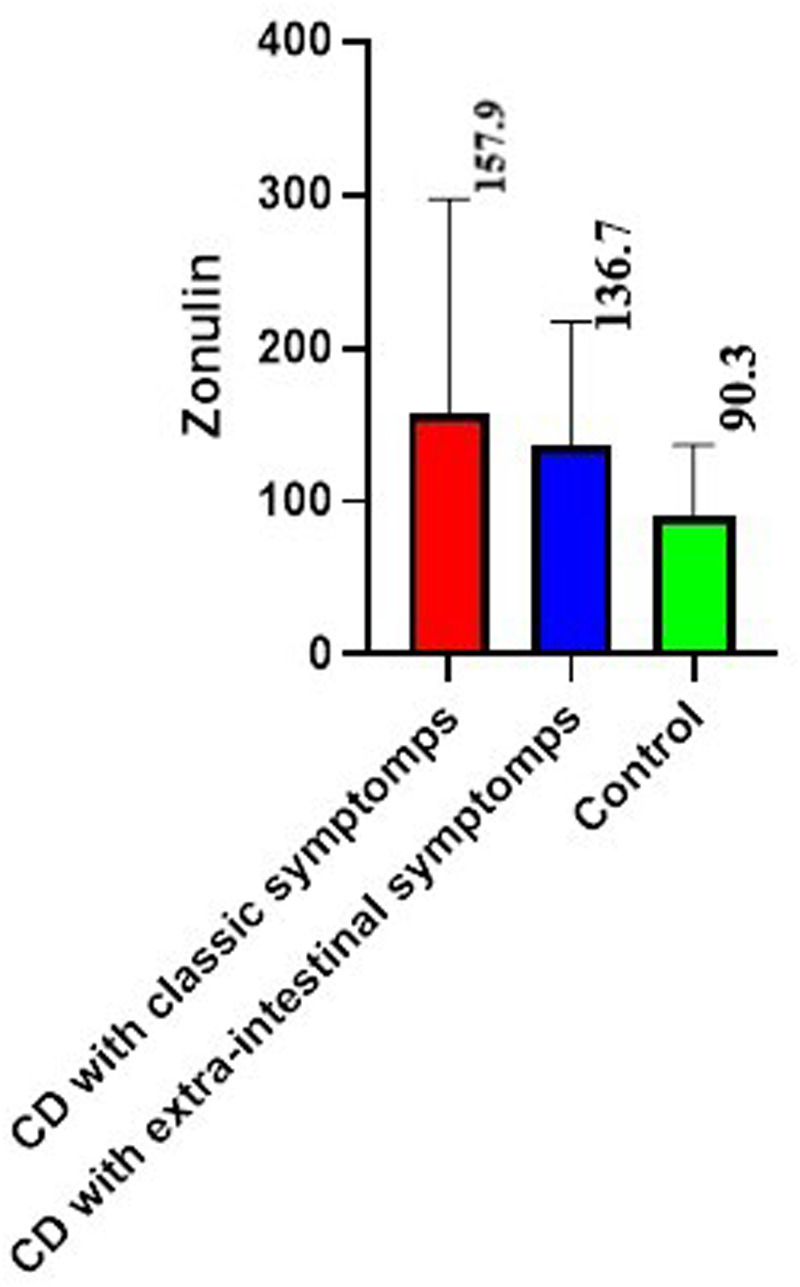
Fecal zonulin level in patients with celiac disease (CD), ng/ml (mean with SD).

The mean value of fecal zonulin in patients with CD was 150.5 ± 35,8 ng/ml, which was 2 times higher than the control value 90,3 ± 15,2 ng/ml (*p* < 0.01) (Figure 1).

If we consider the groups separately, among children with classical CD, fecal zonulin was increased in 41.2% (mean value for the group 157.9 ± 29.8 ng/ml (*p* < 0.02 relative to the control), among patients with extraintestinal symptoms in half of patients (mean value for the group 136.7 ± 17.0 ng/ml).

### 3.3 i-FABP levels depending on the form of celiac disease

**Figure 2:**
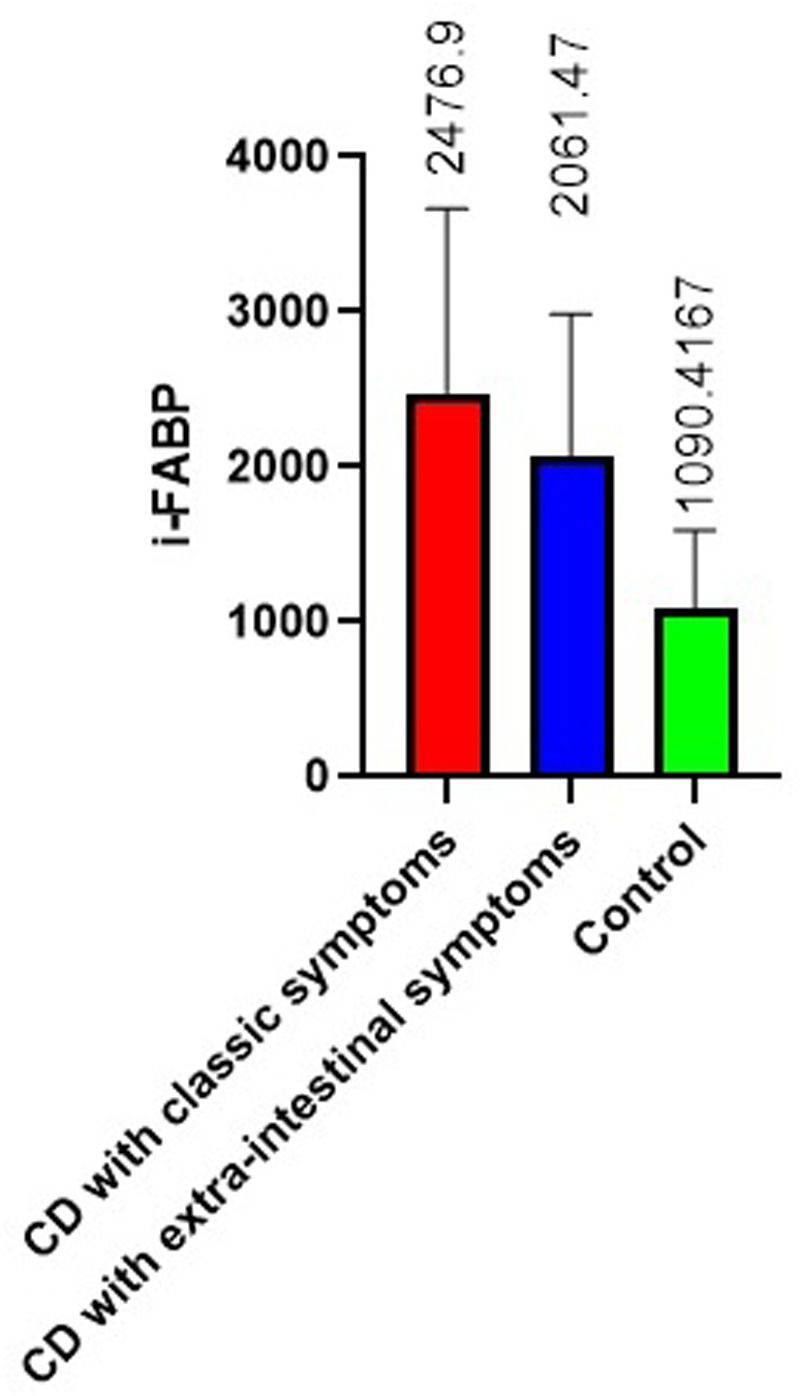
i-FABP level in celiac disease, pg/ml (mean with SD).

The results of measuring the activity of i-FABP in children with CD demonstrated a significant increase in its values compared to the control. The mean i-FABP value in patients was 2293.5 ± 1075.8 pg/ml, which was almost 1.8 times higher than the control value 1090.4 ± 325.8 pg/ml (*p* < 0,0001) (Figure 2).

In the group of patients with classical CD, as well as among children with CD with extraintestinal manifestations, this indicator was increased in half of patients. The mean value in groups was 2476.9 ± 297.4 pg/ml (*p* < 0.001 compared to the control) and 2061.47 ± 291.5 pg/ml (*p* < 0.01 compared to the control, respectively).

### 3.4 Correlation analysis of clinical and laboratory findings and fecal zonulin/I-FABP

Evaluation of the correlations in the group 1 shows a weak positive relationship was found between the level of zonulin and stool frequency (*r* = 0.35, *p* < 0.02), as well as a weak positive relationship with the level of tissue transglutaminase IgA (*r* = 0.36) (Table 2).

**Table 2:** Correlations of clinical and laboratory findings and fecal zonulin/I-FABP in classical CD and CD with extraintestinal manifestations.

Indicator	Fecal zonulin		i-FABP	
	Classic CD	CD with extraintestinal manifestations	Classic CD	CD with extraintestinal manifestations
Stool frequency	0,35	–	0,53	–
tTG-IgA	0,36	–	–	0,58
tTG-IgG	–	0.35	–	–
The number of leukocytes in the blood	0,31	–	0,41	0,52
Weight	–	−0,37	−0,40	−0,34
Height	–	−0,36	−0,36	−0,34
BMI	–	–	−0,40	−0,53
Time of appearance of the first symptoms	–	–	–	−0,37
Time of diagnosis	–	−0,30	−0,28	−0,35
Protein level	–	−0,34	−0,38	–
Histological picture according to Marsh	-	–	–	0,34

CD, celiac disease; I-FABP, intestinal fatty acid binding protein; tTG-IgA, tissue transglutaminase IgA; tTG-IgG, tissue transglutaminase IgG; BMI, body mass index.

More correlation relationships were found in the group 2: a weak inverse correlation relationship between zonulin and weight and height (*r* = −0.37 and *r* = −0.36, *p* < 0.02), a weak positive relationship between zonulin level and antibodies to tissue transglutaminase IgG (*r* = 0.35, *p* < 0.02), a weak negative relationship with protein level (*r* = −0.34, *p* < 0.02), and a weak inverse correlation relationship between zonulin and the time of diagnosis (*r* = −0,30, *p* < 0.02).

When assessing the correlation with i-FABP in the group 1, there was a moderate positive relationship with the stool frequency (*r* = 0.53, *p* < 0.02), a weak positive relationship with the number of leukocytes (*r* = 0.41, *p* < 0.02), and a weak negative relationship was also found between the level of i-FABP and anthropometric indicators (weight, height, BMI)—*r* = −0.40, *r* = −0.36, *r* = −0.40 (*p* < 0.02), respectively. There were also weak negative associations between the level of i-FABP and the time of diagnosis, as well as the level of the protein *r* = −0.28 (*p* < 0.02) and *r* = −0.38 (*p* < 0.02), respectively.

In the group 2, a moderate positive relationship was recorded between the level of i-FABP and antibodies to tissue transglutaminase IgA (*r* = 0.58, *p* < 0.02), as well as with the level of leukocytes (*r* = 0.52, *p* < 0.02), a weak negative relationship between the level of i-FABP and such anthropometric indicators as weight and height: *r* = −0.34 (*p* < 0.02), *r* = −0.34 (*p* < 0.02), respectively, there was a moderate negative relationship with BMI *r* = −0.53 (*p* < 0.02). Weak negative relationships were established between the level of i-FABP and the time of appearance of the first signs, as well as the establishment of the diagnosis: *r* = −0.37 (*p* < 0.02), *r* = −0.35 (*p* < 0.02), respectively. In contrast to the group 1, there was also a weak positive relationship of i-FABP with morphological changes according to Marsh (*r* = 0,34, *p* < 0.02).

## 4 Discussion

This study is the second study to establish fecal zonulin values in the pediatric population up to 16 years of age. The mean fecal zonulin value among healthy children in our study was significantly lower than the data of Gallego et al. (16) and the values presented by Łoniewska et al. (36), who conducted a single study analyzing fecal zonulin in healthy people during the first 2 years of life and observed an increase in fecal levels from birth to 2 years.

iFABP values in plasma of healthy children in our study were 1.3 times higher than the values in Logan et al. study (37), close to the data of Bottasso Arias et al. (28).

In the group of patients with CD, children with the intestinal form of the disease predominated, whose clinical picture was accompanied by severe protein-energy deficiency and pronounced atrophy of the small intestinal mucosa during morphological examination: atrophy of stage 3, according to Marsh, was found in 95% of children, while in patients with CD with extraintestinal manifestations, stage 2, according to Marsh, was detected 1.5 times less often, despite the later diagnosis.

As can be seen from our results, in half of the patients with CD, the level of fecal zonulin was three times higher than the control values. There were differences in fecal zonulin values among children with CD depending on the variant of the disease. Higher numbers were established in children with the classical form of CD. The same pattern is described by Gallego et al. (16). The authors also note a decrease in fecal zonulin levels in children with CD against the background of GFD, and a dependence of its indicators on the duration of the diet is noted.

Statistically significant differences in zonulin values between patients with CD and the control group emphasize the pronounced intestinal permeability in the active phase of the disease, which is consistent with the data of other researchers (16, 33, 38).

The data are worthy of attention (39) that children with CD showed a significant increase in zonulin levels during 18.3 months (range 6–78 months) preceding the development of the disease. The obtained data suggest that fecal zonulin can be used as a biomarker for preclinical screening of CD.

Statistically significant differences in i-FABP values between patients with CD and the control group highlight the presence of severe enterocyte damage.

As it has been noted in several studies, the level of i-FABP in blood plasma in patients with CD is higher than in healthy people at diagnosis, which indicates damage to the mucosa (20, 21, 24, 26, 29–32). Moreover, it has been suggested that patients who meet the four criteria for diagnosing CD (clinical presentation, tTG-IgA levels above 10 U/ml and IgA-EMA positivity, HLA-DQ2 and/or DQ8 genotype), together with increased serum i-FABP levels, may be diagnosed without a biopsy (24). In addition, i-FABP levels may be useful for disease monitoring from the onset of GFD treatment, as they correlate with intestinal injury and repair (24).

Retrospective studies have shown a significant correlation between serum i-FABP levels in pediatric patients with CD and Marsh histological values at the time of diagnosis (21). In our studies, we found only a weak association between i-FABP values and histological mucosal lesions in children with extraintestinal manifestations of CD.

Similar results were obtained by Israeli scientists (40). In the group of patients with CD, there was a higher level of I-FABP upon confirmation of CD compared to the control group (median 641.7 pg/ml vs. 334 pg/ml; *p* < 0.05). I-FABP levels differed significantly between patients whose tTG-IgA level was 3–10 times the upper limit of normal (ULN) compared to patients with values >10 times ULN (median 432.2 pg/ml vs. 796.2 pg/ml; *p* < 0.05). In patients with CD, a significant decrease in the median i-FABP level was observed after 6 months of GFD.

The analysis of correlational relationships in our study demonstrated the most significant relationship between i-FABP indicators and stool frequency in the classic form of the disease, and an inverse relationship with BMI and values of antibodies to tissue transglutaminase in CD with extraintestinal manifestations. Fecal zonulin values had a weak association with antibodies to tissue transglutaminase and stool frequency in the classic form of the disease and an inverse relationship with the weight and height of children with extraintestinal manifestations.

## 5 Conclusions

Thus, our study demonstrated a relationship between the clinical manifestations of CD and the levels of fecal zonulin and i-FABP in the blood plasma of children with CD. The growth of fecal zonulin and i-FABP demonstrates a certain relationship between the severity of clinical manifestations and the increase in the values of non-invasive markers of small intestinal damage, also they can serve as markers of permeability and damage of intestinal barrier in CD, which will open up new possibilities for understanding the processes of restoration of the small intestinal mucosa to improve the prognosis (outcome) of the disease.

## Data Availability

The raw data supporting the conclusions of this article will be made available by the authors, without undue reservation.

## References

[1] Lindfors K Ciacci C Kurppa K Lundin KEA Makharia GK Mearin ML Coeliac disease. Nat Rev Dis Prim. (2019) 5:3–10. 10.1038/s41572-018-0054-z 30631077

[2] Singh P Arora A Strand TA Leffler DA Catassi C Green PH Global prevalence of celiac disease: systematic review and meta-analysis. Clin Gastroenterol Hepatol. (2018) 16:823–36. 10.1016/j.cgh.2017.06.037 29551598

[3] Kamilova AT Azizova GK Poddighe D Umarnazarova ZE Abdullaeva DA Geller SI Celiac disease in Uzbek children: insights into disease prevalence and clinical characteristics in symptomatic pediatric patients. Diagn. (2023) 13:3066–72. 10.3390/diagnostics13193066 PMC10572208 37835809

[4] Roslavtseva EA Pakhomovskaya NL Borovik TE Potapov AS Khomeriki SG. Atypical celiac disease: a clinical case. Ped Pharm. (2012) 9(4):81–5. (In Russ). 10.15690/pf.v9i4.397

[5] Kamilova AT Azizova GK Geller SI. Current state of celiac disease diagnosis in Uzbekistan: problems and solutions. Vopr det Dietol (Pediatr Nutr). (2021) 19(4):15–22. (In Russ). 10.20953/1727-5784-2021-4-15-22

[6] Zybina NN Nikonov EL Gershtein ES Memdli ZZ Stilidi IS Kushlinskii NE. Zonulin is a marker of epithelial and endothelial barrier functions in non-communicable diseases (narrative review). Russ J of Evid Bas Gastroent. (2022) 11(1):28–44. (In Russ). 10.17116/dokgastro20221101128

[7] Szymanska E Wierzbicka A Dadalski M Kierkus J. Fecal zonulin as a noninvasive biomarker of intestinal permeability in pediatric patients with inflammatory bowel diseases—correlation with disease activity and fecal calprotectin. J Clin Med. (2021) 10:3905–10. 10.3390/jcm10173905 34501351 PMC8432014

[8] Binienda A Twardowska A Makaro A Salaga M. Dietary carbohydrates and lipids in the pathogenesis of leaky gut syndrome: an overview. Int J Mol Sci. (2020) 21:8368–73. 10.3390/ijms21218368 33171587 PMC7664638

[9] Fasano A Not T Wang W Uzzau S Berti I Tommasini A Zonulin, a newly discovered modulator of intestinal permeability, and its expression in coeliac disease. Lancet. (2000) 355(9214):1518–19. 10.1016/S0140-6736(00)02169-3 10801176

[10] Khavkin AI. Microflora of the Digestive Tract. Moscow: Social Pediatrics Fund (2006). p. 416.

[11] Zhang YG Xia Y Lu R Sun J. Inflammation and intestinal leakiness in older HIV+ individuals with fish oil treatment. Genes Dis. (2018) 5(3):220–25. 10.1016/j.gendis.2018.07.001 30320186 PMC6176151

[12] Pastor L Langhorst J Schröder D Casellas A Ruffer A Carrillo J Different pattern of stool and plasma gastrointestinal damage biomarkers during primary and chronic HIV infection. PLoS One. (2019) 14(6):e0218000. 10.1371/journal.pone.0218000 31185037 PMC6559643

[13] Linsalata M Riezzo G D’Attoma B Clemente C Orlando A Russo F. Noninvasive biomarkers of gut barrier function identify two subtypes of patients suffering from diarrhoe a predominant-IBS: a case-control study. BMC Gastroenterol. (2018) 18(1):167–73. 10.1186/s12876-018-0888-6 30400824 PMC6219148

[14] Moser AM Spindelboeck W Halwachs B Strohmaier H Kump P Gorkiewicz G Effects of an oral synbiotic on the gastrointestinal immune system and microbiota in patients with diarrhea-predominant irritable bowel syndrome. Europ J of Nutr. (2018) 58(7):2767–78. 10.1007/s00394-018-1826-7 PMC6768888 30251020

[15] Khasanova SS Kamilova AT. Dynamics of fecal zonulin values in premature infants of the first two weeks of life. Ros Vestn Perinatol I Pediatr. (2019) 64:(2):52–6. 10.21508/1027-4065-2019-64-2-52-56

[16] Martínez Gallego MÁ Crespo Sánchez MG Serrano Olmedo MG Buño Soto A Álvarez Casasempere S Nozal P Trends in faecal zonulin concentrations in paediatric patients with celiac disease at baseline and on a gluten-free diet: exploring correlations with other faecal biomarkers. Nutrients. (2024) 16:684–94. 10.3390/nu16050684 38474812 PMC10934140

[17] Levy E Ménard D Delvin E Montoudis A Beaulieu JF Mailhot G Localization, function and regulation of the two intestinal fatty acid-binding protein types. Histochem Cell Biol. (2009) 132:351–67. 10.1007/s00418-009-0608-y 19499240

[18] Ho SS Wall C Gearry RB Keenan J Day AS. A pilot study evaluating novel urinary biomarkers for crohn’s disease. Inflamm Intest Dis. (2020) 5:212–19. 10.1159/000510682 33313074 PMC7706507

[19] Oldenburger IB Wolters VM Kardol-Hoefnagel T Houwen RHJ Otten HG. Serum intestinal fatty acid–binding protein in the noninvasive diagnosis of celiac disease. APMIS. (2018) 126:186–90. 10.1111/apm.12800 29383769

[20] Adriaanse MP Tack GJ Passos VL Damoiseaux JG Schreurs MW van Wijck K Serum I-FABP as marker for enterocyte damage in coeliac disease and its relation to villous atrophy and circulating autoantibodies. Aliment Pharmacol Ther. (2013) 37:482–90. 10.1111/apt.12194 23289539

[21] Vreugdenhil AC Wolters VM Adriaanse MP Van den Neucker AM van Bijnen AA Houwen R Additional value of serum I-FABP levels for evaluating celiac disease activity in children. Scand J Gastroenterol. (2011) 46:1435–41. 10.3109/00365521.2011.627447 22029621

[22] Wiercinska-Drapalo A Jaroszewicz J Siwak E Pogorzelska J Prokopowicz D. Intestinal fatty acid binding protein (I-FABP) as a possible biomarker of ileitis in patients with ulcerative colitis. Regul Pept. (2008) 147:25–8. 10.1016/j.regpep.2007.12.002 18201778

[23] Al-Saffar AK Meijer CH Gannavarapu VR Hall G Li Y Diaz Tartera HO Parallel changes in harvey-bradshaw index, TNFa, and intestinal fatty acid binding protein in response to infliximab in Crohn’s disease. Gastroenterol Res Pract. (2017) 2017:1745918. 10.1155/2017/17459.18 29201046 PMC5672611

[24] Adriaanse MPM Mubarak A Riedl RG Ten Kate FJW Damoiseaux JGMC Buurman WA Progress towards non-invasive diagnosis and follow-up of celiac disease in children; a prospective multicentre study to the usefulness of plasma I-FABP. Sci Rep. (2017) 7:8671–78. 10.1038/s41598-017-07242-4 28819290 PMC5561259

[25] Uhde M Ajamian M Caio G De Giorgio R Indart A Green PH Intestinal cell damage and systemic immune activation in individuals reporting sensitivity to wheat in the absence of coeliac disease. Gut. (2016) 65:1930–37. 10.1136/gutjnl-2016-311964 27459152 PMC5136710

[26] Adriaanse MP Leffler DA Kelly CP Schuppan D Najarian RM Goldsmith JD Serum I-FABP detects gluten responsiveness in adult celiac disease patients on a short-term gluten challenge. Am J Gastroenterol. (2016) 111:1014–22. 10.1038/ajg.2016.162 27185075

[27] Bodelier AG Pierik MJ Lenaerts K de Boer E Olde Damink SW Hameeteman WM Plasma intestinal fatty acid-binding protein fails to predict endoscopic disease activity in inflammatory bowel disease patients. Eur J Gastroenterol Hepatol. (2016) 28:807–13. 10.1097/MEG.0000000000000616 26919325

[28] Bottasso Arias NM García M Bondar C Guzman L Redondo A Chopita N Expression pattern of fatty acid binding proteins in celiac disease enteropathy. Mediators Inflamm. (2015) 2015:738563. 10.1155/2015/738563 26346822 PMC4540995

[29] Derikx JP Vreugdenhil AC Van den Neucker AM Grootjans J van Bijnen AA Damoiseaux JG A pilot study on the noninvasive evaluation of intestinal damage in celiac disease using I-FABP and L-FABP. J Clin Gastroenterol. (2009) 43:727–33. 10.1097/MCG.0b013e31819194b0 19359998

[30] Ho SSC Keenan JI Day AS. The role of gastrointestinal-related fatty acid-binding proteins as biomarkers in gastrointestinal diseases. Dig Dis Sci. (2020) 65:376–90. 10.1007/s10620-019-05841-x 31529416

[31] Bykova SV Sabelnikova EA Novikov AA Baulo EV Khomeriki SG Parfenov AI. Zonulin and I-FABP are markers of enterocyte damage in celiac disease. Ter Arkhiv. (2022) 94:511–16. 10.26442/00403660.2022.04.201480 36286801

[32] Gandini A De Maayer T Munien C Bertrand K Cairns R Mayne A Intestinal fatty acid binding protein (I-FABP) and CXC3L1 evaluation as biomarkers for patients at high-risk for coeliac disease in Johannesburg, South Africa. Cytokine. (2022) 157:155–65. 10.1016/j.cyto.2022.155945 35841826

[33] Bykova SV Sabelnikova EA Novikov AA The role of non-invasive markers of enterocyte damage and increased permeability in the pathogenesis of celiac disease. Effective Pharm. (2021) 17(4):68–75. 10.33978/2307-3586-2021-17-4-68-75

[34] WHO Multicentre Growth Reference Study (MGRS). (2006).

[35] Oberhuber G Granditsch G Vogelsang H. The histopathology of coeliac disease:time for a standardized report scheme for pathologists. Eur J Gastroenterol Hepatol. (1999) 11(10):1185–94. 10.1097/00042737-199910000-00019 10524652

[36] Łoniewska B Adamek K Wegrzyn D Kaczmarczyk M Skonieczna-Ż ydecka K Clark J Analysis of faecal zonulin and calprotectin concentrations in healthy children during the first two years of life. An observational prospective cohort study. J Clin Med. (2020) 9:777. 10.3390/jcm9030777 32178435 PMC7141325

[37] Logan M MacKinder M Clark CM Kountouri A Jere M Ijaz UZ Intestinal fatty acid binding protein is a disease biomarker in paediatric coeliac disease and Crohn’s disease. BMC Gastroenterol. (2022) 22:260–70. 10.1186/s12876-022-02334-6 35606704 PMC9125891

[38] Lammers KM Lu R Brownley J Lu B Gerard C Thomas K Gliadin induces an increase in intestinal permeability and zonulin release by binding to the chemokine receptor CXCR3. Gastroenterol. (2008) 135:194–204.e3. 10.1053/j.gastro.2008.03.023 PMC2653457 18485912

[39] DaFonte TM Valitutti F Kenyon V Locascio JJ Montuori M Francavilla R Zonulin as a biomarker for the development of celiac disease. Pediatrics. (2024) 153(1):e2023063050. 10.1542/peds.2023-063050 38062791 PMC10754681

[40] Hoofien A Guz-Mark A Zevit N Tsadok Perets T Assa A Layfer O Intestinal fatty acid binding protein levels in pediatric celiac patients in transition from active disease to clinical and serological remission. JPGN Rep. (2021) 2(2):e070. 10.1097/PG9.0000000000000070 37207053 PMC10191526

